# Exploring the effects of health behaviors and mental health on students’ academic achievement: a cross-sectional study on lebanese university students

**DOI:** 10.1186/s12889-023-16184-8

**Published:** 2023-06-26

**Authors:** Dalal Hammoudi Halat, Souheil Hallit, Samar Younes, Mira AlFikany, Sanaa Khaled, Maha Krayem, Sami El Khatib, Mohamad Rahal

**Affiliations:** 1 Academic Quality Department, QU Health, Qatar University, Doha, Qatar; 2 School of Pharmacy, Lebanese International University, Bekaa, Lebanon; 3 School of Medicine and Medical Sciences, Holy Spirit University of Kaslik, P.O. Box 446, Jounieh, Lebanon; 4 Applied Science Research Center, Applied Science Private University, Amman, Jordan; 5 Department of Research, Psychiatric Hospital of the Cross, Jal Eddib, Lebanon; 6 Department of Biomedical Sciences, School of Arts and Sciences, Lebanese International University, Bekaa, Lebanon; 7 Center for Applied Mathematics and Bioinformatics (CAMB), Gulf University for Science and Technology (GUST), Hawally, Kuwait

**Keywords:** Academic achievement, Diet, Eating habits, Physical activity, University students, Lebanon

## Abstract

**Background:**

High academic achievement, an important determinant of future success, is known to be influenced by many factors including dietary behavior, lifestyle and mental health, among others. The objectives of the current study were to explore university students’ nutritive habits, daily lifestyle, and mental status, and to scrutinize the associations between these factors and students’ academic achievement.

**Methods:**

A cross-sectional study was conducted among students of a private Lebanese university, using an electronic survey. Diet, eating habits, physical activity, sleep, and smoking were evaluated, and mental health was assessed using a validated Arabic version of the combined Depression, Anxiety, Stress Score (DASS-8). Academic achievement was measured using the Subjective Academic Achievement Scale (SAAS).

**Results:**

A total of 1677 students participated in the questionnaire. The results of a linear regression taking the SAAS score as the dependent variable, showed that students who have a non-scientific versus scientific major (Beta = 0.53), and having breakfast ≥ 4 days per week compared to less than 2 days (Beta = 0.28) were significantly associated with higher SAAS scores. More psychological distress (Beta = − 0.06) and a higher number of days of eating out (Beta = − 0.07) were significantly associated with lower SAAS scores.

**Conclusions:**

This is the first investigation on a Lebanese university students’ academic success in relation to lifestyle and mental profiles. Better academic achievement was demonstrated by students having healthier dietary and lifestyle habits, as well as less distressing mental status. Such results, in light of the compounded and unprecedented crises with which Lebanon has been assailed, suggest the need to focus on promoting healthy habits among students in higher education as a possible driver of better academic success.

## Background

Academic achievement, a construct crucial for the future success of students, is defined as the level to which students attain predetermined educational goals, or the extent towards which they broadly meet their academic outcomes [1]. It can be assessed by a variety of methods, most commonly the examination scores and the grades assigned by teachers [2], although alternative measures and scales are finding their way for additional prediction of academic success [3]. According to research, students who achieve higher academic success during university coursework are more likely to get employment, to be better paid, to be more efficient at work, and to move more actively and dynamically towards their future [4]. Several factors affect academic achievement, including personal student characteristics (motivation, perception of wellbeing, quality of life, and involvement in activities), learning environment attributes (human resources, class size, teaching styles, rewards, extra-curricular activities, use of technology, evaluation systems, and physical accommodations), family support (home environment, parental support, and family size), and community facilities (clubs, gyms, gatherings, and outdoor events) [5–11].

While the unique period of transition from home or school to university is a time for excitement and liberation [12], it is also typified by inevitable change, multiple challenges, and critical developmental adaptation as students settle into university life and thrive as higher education learners [13, 14]. Being a stage marked by increased independence and autonomy, university life appears to be a fundamental factor which helps adopting healthy habits that affect overall health and mental well-being [15, 16]. Some studies have been carried out to evaluate eating habits and dietary intake in the Middle East and Arab countries. For instance, a study conducted at King Abdulaziz University showed that university students who smoked, rented housing, lived alone, had divorcing parents, and did not frequently exercise had considerably poorer eating habits scores [17]. Furthermore, the prevalence of unhealthy dietary behaviors among adolescents in Morocco was concerning, where many participants skipped breakfast, had inadequate intake of fruits and vegetables, consumed soft drinks, and ate fast foods [18]. Additionally, eating habits in Syria are shifting towards westernized patterns which are rich in carbohydrates, fats, and meat, along with a rise in sedentary lifestyle [19]. In Lebanon, university students have diverse eating habits that reflect a blend of traditional Lebanese cuisine and global influences. While many students maintain a strong connection to their traditional cuisine, which emphasizes fresh vegetables, grains, legumes, lean meats, and olive oil, they also embrace fast food and Western-style cafés available on and around campuses [20, 21]. Snacking culture is also prevalent among students, with frequent indulgence in chips, chocolates, and energy drinks [22, 23]. Substantial evidence suggests associations between health behaviors of the university students and their academic achievement [24, 25], and may be explained by the positive effects of balanced lifestyle on cognitive behavior, brain function, and memory [26, 27]. For example, unhealthy diet, typical of university students due to time restraints, stress, unhealthy snacks, convenience of high-calorie food, cost of healthy food, and easy access to junk food [28], is found to have far reaching repercussions beyond health, and may affect academic performance [29]. According to previous results of a systematic review, dietary habits characterized by regular breakfast intake, lower consumption of energy-dense, nutrient-poor foods, and overall diet quality have positive associations on academic outcomes [30]. Furthermore, in a longitudinal analysis of dietary habits, the shift towards protein-rich food, fruits, and vegetables was associated with a notable increase in academic achievement [31]. Moreover, compared to students with healthy, nutritious, in-home snacking behaviors, adolescents consuming unhealthy snacks had significantly lower Grade Point Average (GPA) [32]. Studies investigating the link between unhealthy diet and cognition have demonstrated that acute consumption of a high-fat diet primes potentiated neuroinflammatory responses and promotes the generation of oxidative stress, which may cause memory deficits and affect brain function [33, 34].

Apart from diet, smoking is another common behavior among university students and is linked to psychological and cognitive effects [35]. Its incidence is predicted by a lack of coping resources, poor health attitudes, and lack of knowledge about smoking consequences on health [36]. Smoking is also linked to low academic performance [37, 38], with students who academically perform better being less likely to smoke, while those with weak performance smoking more often [39]. Short sleep times were also associated with poor academic achievement [40]. University students tend to experience sleep irregularity and problems with sleep quantity and quality, at a phase of life critical for maturity, psychosocial development, and academic attainment [24]. In a systematic review and meta-analysis of sleep disruption in medical students, sleep rhythm and impairment were found to severely affect learning ability and grades, [41]. This calls for the need to improve sleep quality to maintain academic success, perhaps by adhering to consensus guidelines of an average of 7 h of sleep for adults as well as adequate sleep quality [42]. Another health factor related to academic performance is physical activity, with physically active students being more likely to have both physical and mental health benefits [43]. In a study among dental students, physical activity showed a better academic performance with a higher resistance against low GPA [44]; likewise, Al-Drees and Colleagues showed significant positive associations between physical activity and high GPA among medical students [45], promoting the need to establish effective strategies to kindle physical activity during the years of higher education.

In terms of mental health, listed by the World Health Organization among the leading causes of disability globally [46], this issue receives extensive societal attention, with university students expected to have a good mental health and high levels of contentment [47]. Nevertheless, it is reported that university students have higher depression and anxiety rates compared to the general population [48–50], and can be affected by various social, academic, interpersonal, and environmental pressures [51]. Studies showed that students with more appropriate mental health status will have higher level of motivation in their education, contributing to more success [52].

While evidence exists to support the association of health behaviors and mental wellbeing with academic achievement, and to our knowledge, no previous studies have examined such correlations among university students in Lebanon. A country well known for its once famous, top-tier, and robust higher education system in the Middle East, Lebanon is currently facing an intense political, economic, financial, and health crises, aggravated by the COVID-19 pandemic and the explosion of Beirut port on August 4th, 2020 [53, 54]. This dire reality has affected university education and university students lifestyle [50, 55], as well as their mental health [56]. The objective of the current study was to explore university students’ health behaviors including diet, physical activity, sleep, and smoking, as well as their psychological distress, and to shed a light on the correlations between these factors and academic achievement.

## Methods

### Study design

This study was a cross-sectional study conducted among students of the Lebanese International University, which has nine campuses distributed over the eight Lebanese governorates, and a student population of about 35,000 students [57]. The study was conducted using an electronic survey via Google Forms and sent to all enrolled students who were registered in the fall semester during the academic year 2022–2023 via their institutional email. Excluded were students who refused to participate. The survey was prepared in Arabic language, the native language of all of the surveyed participants and the survey link was accessible from December 15th, 2022 till January 5th, 2023, with few reminders regularly sent to increase the number of collected responses. The purpose and the scope of the study were explained in the introductory statement of the survey, and the target students were informed that their participation in the study was voluntary and remains without associated risks. They were also assured that their responses would remain anonymous and confidential. The completion of the entire survey was considered as informed consent on behalf of the student to participate.

The conduct and reporting of this study follow the statement of the Strengthening the Reporting of Observational Studies in Epidemiology (STROBE) guidelines [58].

### Minimal sample size calculation

G*Power software was used to determine the sample size. The minimum required sample size was 600 participants, considering an alpha error of 5%, a power of 95%, a minimal model R-square of 5% and 20 predictors allowed to be included in the model.

### Survey instrument

The survey consisted of four sections. The first section addressed sociodemographic characteristics of the participants as well as their major and year of study, internet availability, and monthly income at their household. The second section addressed body mass index (BMI) and lifestyle habits. These latter included number of week days at which the student had breakfast, the number and the type of consumed snacks, smoking cigarettes and/or waterpipe, weekly average of eating out, daily fluid intake and sleep hours [17, 21, 28, 59–61]. In addition, a validated scale for measuring physical activity was used [62].

To assess academic achievement, the third section of the survey used the Subjective Academic Achievement Scale (SAAS) by Stadler and Colleagues [63]. The SAAS consisted of five items, which participants answer on a “yes”-or-“no” basis, where the number of affirmative (yes) responses were counted towards a score ranging from zero to 5. Higher scores were considered to reflect a better academic achievement (McDonald’s omega = 0.73).

Finally, the fourth part of the survey addressed mental health of students by using a validated Arabic version of the combined Depression, Anxiety, Stress Score composed of 8 items (DASS-8) [64] (McDonald’s omega = 0.86).

### Statistical analysis

IBM SPSS Statistics for Windows, version 25.0 (IBM Corp., Armonk, N.Y., USA) was used to perform the data analysis. McDonald’s omega values were computed for reliability analysis. The SAAS score was considered normally distributed, with its skewness and kurtosis values falling between − 1 and + 1 [65]. The Student t and ANOVA tests were used to compare two means and three or more means respectively. Pearson test was used to correlate two continuous variables. Bonferroni correction was applied to take into consideration multiple analysis; the corrected *p* value was estimated at 0.002, after dividing 0.05 by the total number of variables in the analysis (= 24). A linear regression was conducted, taking the SAAS score as the dependent variable. Factors that showed a significant *p* in the bivariate analysis were entered as independent variables in the final model. *P* < .05 was considered statistically significant.

## Results

A total of 1677 students filled the survey. Their mean age was 20.99 ± 3.92, with 71.7% females. The mean BMI was 23.16 ± 4.81, with 471 (28.2%) of participants being overweight/obese. All details about their lifestyle behaviors (eating habits, smoking, sleeping) and psychological distress are summarized in Table 1.

**Table 1:** Sociodemographic and other characteristics of the students (n = 1677)

	n (%)
Sex	
Male	475 (28.3%)
Female	1202 (71.7%)
Nationality	
Lebanese	1417 (84.5%)
Non-Lebanese	260 (15.5%)
Governorate	
Beirut	257 (15.4%)
Mount Lebanon	215 (12.8%)
North/Akkar	302 (18.0%)
South/Nabatiyeh	522 (31.1%)
Bekaa/Baalbeck-Hermel	381 (22.7%)
Major	
Scientific*	1341 (80.0%)
Non-scientific**	336 (20.0%)
Family monthly income (in LBP)	
Low (< 5 million)	555 (33.1%)
Intermediate (5–10 million)	623 (37.1%)
High (10–15 million)	261 (15.6%)
Very high (> 15 million)	238 (14.2%)
Breakfast per week	
<2 days	290 (17.3%)
2–3 days	463 (27.6%)
≥4 days	924 (55.1%)
Snack- vegetables and fruits	
No	416 (24.8%)
Yes	1261 (75.2%)
Snack- Desserts and carbonated beverages	
No	455 (27.1%)
Yes	1222 (72.9%)
Snack- Fat	
No	781 (46.6%)
Yes	896 (53.4%)
Snack- Carbohydrates	
No	452 (27.0%)
Yes	1225 (73.0%)
Snack- Milk products	
No	925 (55.2%)
Yes	752 (44.8%)
Snack- Other	
No	838 (50.0%)
Yes	838 (50.0%)
Cigarette smoking	
No	1489 (88.8%)
Yes	188 (11.2%)
Waterpipe smoking	
No	1181 (70.4%)
Yes	496 (29.6%)
Hours of sleep per day	
<7	774 (46.2%)
7–8	713 (42.5%)
>8	190 (11.3%)
	**Mean ± SD**
Age, years	20.99 ± 3.92
Body Mass Index, kg/m^2^	23.16 ± 4.81
Number of snacks per day	2.18 ± 1.04
Number of cigarettes per day	1.38 ± 5.17
Number of waterpipes per week	1.58 ± 3.62
Number of days eating out	1.38 ± 1.34
L of fluids per day	1.95 ± 2.24
Physical activity index	7.75 ± 2.69
Psychological distress	10.04 ± 6.32
Academic performance	3.71 ± 1.44

*Scientific majors included: pharmacy, biomedical sciences, and biology.

**Non-scientific majors included: arts, education, engineering, and business.

### Bivariate analysis

The results of the bivariate analysis are summarized in Tables 2 and 3. A higher mean SAAS score (better academic achievement) was seen in students who have a non-scientific vs. scientific major, in those who have breakfast ≥ 4 days per week, in those who have snacks composed of vegetables and fruits. In addition, a higher number of waterpipes per week, a higher number of days of eating out and more psychological distress were significantly associated with lower SAAS scores, whereas a higher physical activity index was significantly associated with higher SAAS scores. Moreover, a higher BMI was associated with lower academic achievement.

**Table 2:** Bivariate analysis of categorical factors associated with SAAS.

	Mean ± SD	*P*
Sex		0.957
Male	3.71 ± 1.36	
Female	3.71 ± 1.48	
Nationality		0.025
Lebanese	3.74 ± 1.43	
Non-Lebanese	3.53 ± 1.49	
Major		**< 0.001**
Scientific	3.63 ± 1.47	
Non-scientific	4.03 ± 1.29	
Family monthly income (in LBP)		0.115
Low (< 5 million)	3.61 ± 1.45	
Intermediate (5–10 million)	3.71 ± 1.48	
High (10–15 million)	3.78 ± 1.41	
Very high (> 15 million)	3.87 ± 1.36	
Breakfast per week		**< 0.001**
<2 days	3.36 ± 1.54	
2–3 days	3.62 ± 1.47	
≥4 days	3.87 ± 1.38	
Snack- vegetables and fruits		**< 0.001**
No	3.44 ± 1.60	
Yes	3.80 ± 1.38	
Snack- Desserts and carbonated beverages		0.090
No	3.81 ± 1.41	
Yes	3.67 ± 1.45	
Snack- Fat		0.681
No	3.70 ± 1.47	
Yes	3.72 ± 1.42	
Snack- Carbohydrates		0.263
No	3.77 ± 1.39	
Yes	3.69 ± 1.46	
Snack- Milk products		0.486
No	3.69 ± 1.47	
Yes	3.74 ± 1.41	
Snack- Other		0.183
No	3.76 ± 1.43	
Yes	3.66 ± 1.46	
Cigarette smoking		0.026
No	3.74 ± 1.43	
Yes	3.47 ± 1.55	
Waterpipe smoking		0.004
No	3.78 ± 1.41	
Yes	3.55 ± 1.51	
Hours of sleep per day		0.390
<7	3.68 ± 1.46	
7–8	3.76 ± 1.41	
>8	3.63 ± 1.52	

Numbers in bold indicate significant *p* values after Bonferroni correction.

**Table 3:** Correlation matrix of continuous variables

	1	2	3	4	5	6	7	8	9	10
1. SAAS	1									
2. BMI	− 0.05*	1								
3. Number of snacks per day	− 0.01	0.01	1							
4. Number of cigarettes per day	− 0.07**	0.06*	− 0.03	1						
5. Number of waterpipes per week	− 0.09***	0.08**	0.02	− 0.02	1					
6. Number of days eat out	− 0.09***	0.01	0.11***	0.15***	0.21***	1				
7. Liters of fluids per day	0.02	0.05*	0.04	0.04	0.001	0.02	1			
8. Physical activity	0.08**	0.09***	0.05	0.09**	− 0.07**	0.03	0.14***	1		
9. Psychological distress	− 0.27***	0.07**	0.02	0.01	0.09***	0.03	− 0.04	− 0.16***	1	

*p < .05; **p < .01; ***p < .001

### Multivariable analysis

The results of a linear regression taking the SAAS score as the dependent variable, showed that students who have a non-scientific vs. scientific major (Beta = 0.53), and having breakfast ≥ 4 days per week compared to less than 2 days (Beta = 0.28) were significantly associated with higher SAAS scores. Finally, more psychological distress (Beta = − 0.06) and a higher number of days of eating out (Beta = − 0.07) were significantly associated with lower SAAS scores (Table 4).

**Table 4:** Linear regression taking the SAAS score as the dependent variable

	Unstandardized Beta	Standardized Beta	*p*	95% CI
Major (nonscientific vs. scientific*)	0.53	0.15	**< 0.001**	0.37; 0.70
Snack- vegetables and fruits (yes vs. no*)	0.17	0.05	**0.033**	0.01; 0.33
Breakfast per week (2–3 days vs. < 2 days*)	0.14	0.04	0.191	− 0.07; 0.34
Breakfast per week (≥ 4 days vs. < 2 days*)	0.28	0.10	**0.003**	0.09; 0.47
Number of waterpipes per week	− 0.02	− 0.04	0.093	− 0.04; 0.003
Number of days eating out	− 0.07	− 0.06	**0.008**	− 0.12; − 0.02
Physical activity	0.02	0.03	0.184	− 0.01; 0.04
Psychological distress	− 0.06	− 0.26	**< 0.001**	− 0.07; − 0.05

*Reference group; Nagelkerke R^2^ = 0.112; numbers in bold indicate significant *p* values.

## Discussion

To the best of our knowledge, this is the first study to be conducted among Lebanese university students with the aim of examining the correlations between health behaviors/customs (diet, physical activity, sleep, and smoking) and psychological distress with academic achievement.

Our results showed better academic achievement denoted by a higher mean SAAS score among university students who have a non-scientific major compared to those with scientific major. This result is contrary to the findings of other studies. For instance, Tadese et al. reported that medical/health science students were more likely to achieve good academic points in comparison to non-medical/non-health students [66]. Furthermore, academic performance was significantly higher among health science students in Southern Ethiopia [67]. The possible explanation of our result might be that students enrolled in scientific majors might have higher levels of stress because of their exams, assignments, and other duties during their years of study than those in non-scientific majors, and this has mostly affected their lower SAAS score. Since the SAAS score is subjective, students may have evaluated their academic success in relation to their own exerted effort, personal goals, and the achievement of their peers. Moreover, only around 20% of the respondents are enrolled in non-scientific majors and as such they may not represent the whole population, perhaps influencing our results. It may be tempting to explore the relation between study major and SAAS more diligently, using a larger non-scientific major student sample, and perhaps including less subjective methods of achievement like test grades or GPA.

Furthermore, the present study demonstrated a higher mean SAAS score in students who have breakfast ≥ 4 days per week, as well as those who have snacks composed of vegetables and fruits. This is consistent with studies conducted on adolescents and university students, which found that eating breakfast more frequently was favorably related to academic achievement [25, 68–70]. Findings of a study conducted on a sample of Australian students also showed that skipping breakfast on a daily basis was significantly associated with lower GPA [4]. Additionally, a study performed by Verulava et al. revealed that academic performance of students in Georgia had a statistically significant association with the quality of breakfast, as well as the frequency and diversity of fruit consumption [71]. Eating breakfast can enhance students’ cognitive abilities, improve memory, and increase attention span during the learning process; in contrast, skipping breakfast will negatively impact students’ psychological and physical growth in addition to their academic performance [72, 73]. Concerning the intake of healthy snacks composed of vegetables and fruits, this finding is in line with a systematic review on the relationship between eating habits and academic success for university students, which looked at seven different studies, and found that five of those reported higher academic achievement with increased fruit intake [68]. However, even though some studies found that university students who consumed more fruits and vegetables performed better academically, it was also found that the vast majority of undergraduates do not consume the required daily intake of fruits and vegetables [74–77]. On the other hand, our study found that students who reported a higher number of days of eating out had a lower SAAS score. Two possible explanations can be drawn from this finding: It could be hypothesized that students who spend more time outside would have less time for their academic work and may be less efficient in time management in favor of their study; also, those students consume more fast food which is notorious for its deficiency in a range of nutrients [78, 79].

Poor academic performance is known to be correlated with inadequate nutrient intakes, notably of iron, and excessive intakes of fat and added sugar as a result of frequent fast food meal consumption [80]. These findings provide additional support to the recommendations of consuming a diet that offers the amount micro- and macronutrients necessary to support healthy growth and development [81]. A variety of dietary elements have a significant effect on cellular or molecular processes that are essential for maintaining cognitive function. Thus, through controlling synaptic transmission, membrane fluidity, neurotransmitter routes, and signal-transduction pathways, nutrition can impact a number of physiological brain functions [60, 82], and therefore, probably affecting academic success. Furthermore, fruits and vegetables include both flavonoid and non-flavonoid polyphenols, which influence learning and memory by enhancing neuronal signaling and upregulating the production of antioxidant and anti-inflammatory substances [60]. In contrast, a high intake of simple carbohydrates and saturated fats, as those found in ready meals and fast food, raises the impact of oxidative stress and lowers the levels of neurotrophic substances derived from the hippocampus [81].

Moreover, the mean BMI in our study sample of Lebanese university students was 23.16 ± 4.81, which is in line with previously conducted research in Lebanon although students had shown a relatively alarming prevalence of unhealthy dietary practices and lifestyle behaviors [21, 83]. BMI was also within the range of normal among most university students in Egypt [84], Morocco [85], Saudi Arabia [86, 87], and United Arab Emirates [88]. On the other hand, higher values of BMI were observed in some studies indicating a higher prevalence of overweight and obesity among university students compared to the general population. This can be possibly explained by the lifestyle changes associated with the transition to college life, such as increased academic demands, stress, unhealthy eating habits, decreased physical activity, and increased sedentary behavior [89–93]. In our study, there was no association between BMI and academic achievement which is similar to the results of a study conducted in Portugal [94], Saudi Arabia, and India [95, 96]; however, other studies found that high BMI negatively affects academic achievement [97–99]. Additionally, waterpipe smoking appeared to be a more common smoking habit than cigarette smoking (29.6% versus 11.2%) in our sample of students. This finding demonstrates that waterpipe smoking is a viable alternative to cigarette smoking and is growing in popularity among young people [100]. A systematic review revealed an alarmingly high prevalence of cigarette and waterpipe smoking among university students in some Arab countries [101]. Additionally, previously conducted studies in the region showed high prevalence rates of smoking in Lebanon, Jordan and Palestine [102–104]. The acceptance of smoking among families in Arab culture also lends further support to this finding and could reflect an emerging epidemic of waterpipe smoking among youth in the Eastern Mediterranean Region [105]. The current study found that university students who frequently consume a higher number of waterpipes per week had a lower SAAS score than their counterparts, although this was observed only in the bivariate analysis. Our results were overall consistent with other studies that reported lower self-reported academic achievement among students who are current waterpipe and cigarette tobacco smokers [4, 61, 100, 106, 107]. This might be the result of the social network of friends that waterpipe tobacco smoking fosters, which promotes smoking and subtly diverts focus away from studies. Available research indicates a complicated rather than a simple relationship between smoking and academic achievement that interacts with or is mediated by students’ psychosocial context, offering opportunities for further investigation into the factors that influence this relationship [108, 109].

Regarding physical activity, a significant association has been revealed between physical activity and academic achievement, where a higher physical activity index was positively associated with higher SAAS scores in bivariate analysis. Although this result needs careful interpretation, a similar association was illustrated between physical activity and GPA in a cross-sectional study conducted among 409 medical students in Saudi Arabia [110]. Besides, Lipošek et al. found that a duration of 2 to 3 h of physical activity per week were positively correlated with academic success [111]. Other studies reported no association between physical activity and total sedentary time with academic achievement of students [4, 112, 113]. On the basis of the above mentioned facts, it can be concluded that speculative theories about the process by which exercise causes cognitive changes are accurate [114]. Increased levels of neurotrophins and neurotransmitters, improved blood flow to the brain, the development of new brain cells, blood vessels, and the protein brain-derived neurotrophic factor (BDNF), are just a few of the positive effects of physical activity. Alterations in synaptic plasticity and spine density may also play a role in mediating the positive effects of physical activity on learning and memory [115–118].

Concerning the mental health impact on academic achievement, it was found that more psychological distress was significantly associated with lower SAAS scores. This is in line with previous findings that demonstrated poor academic self-perception and greater risk for academic failure and dropout among students with higher psychological distress [119–123]. The transition from adolescence to adulthood that university students pass through causes them to frequently experience various levels of psychological discomfort, which has an impact on their academic performance. Given that they must manage developmental responsibilities including academics, social interactions, and personal needs, it is more likely that many of them would report low psychological well-being that would engender notable implications on their educational and career development [124]. It is also worth mentioning that Lebanese university students, in particular, may be more prone to suffer from mental health problems as a consequence of COVID-19, the Lebanese economic crisis, political instability, the fourth of August Beirut port explosion, and healthcare shortage [125]. For instance, the findings of a previously conducted study on mental health of the Lebanese young population demonstrated that a significant number of respondents showed symptoms of anxiety (42%), insomnia (21.4%), and depression (42.6%), all of which would negatively affect the academic performance of our students irrespective of their health behaviors [126]. Therefore, the wider scientific community and international organizations are urged to provide support for preventing additional detrimental effects on the Lebanese population’s mental health, and most probably, the academic performance of university students.

### Clinical implications

The findings reported herein may call for immediate actions regarding the health and mental correlates of university students. Healthcare providers and researchers, alike, may benefit from these baseline data to (i) promote the concepts of healthy eating habits and general healthy lifestyle among university students, especially in what relates to the effect of such habits on their academic success; (ii) foresee and endorse good mental health and psychological stability in this vulnerable population, given its possible consequences on achievement; and (iii) invest more in research, funding, and focus on these areas.

### Strengths and Limitations

This study is the first in Lebanon to explore the effect of healthy habits and mental status on the academic achievement among university students, thus adding to the body of evidence on this issue. Targeting a nationwide university student sample, assessing different health and mental status factors, and using standardized scales are among the strengths of this study. However, this investigation does have limitations. First, our analysis of academic achievement depended on SAAS, which can be subjective.Plus, this scale has not been validated in Arabic. Using more concrete determinants like grades or GPA, would possibly yield results with lower student bias. Moreover, the surveyed student population derived from one university, while a better envisioning of our data would have come from a more diverse student population. Additionally, assessments conducted by the survey instrument were not free from recall bias which may have affected our results. Accordingly, a more thorough analysis perhaps with the use of interviews and focused questioning on health habits is warranted for future studies.

## Conclusion

In conclusion, the results from this cross-sectional analysis reveal that adopting healthy habits including breakfast, healthy snacks, and less meals outside contribute to better academic achievement among university students. Also, mental distress is more likely to reduce the measures of success in this population. While further research is needed to obtain more robust findings, this preliminary analysis is a call for policymakers and stakeholders in higher education to focus on the need to foster a healthy lifestyle and a mentally acceptable atmosphere at universities. Educators should plan for interventions, activities, and initiatives to promote a combination of healthy lifestyle and mental well-being, due to the influence both have on increasing the odds of higher academic performance among university students.

## Data Availability

The datasets generated and/or analyzed during the current study are not publicly available due to limitations of ethical approval involving the participants’ data and anonymity but are available from the corresponding authors on reasonable request.

## References

[1] Jayanthi SV, Balakrishnan S, Ching ALS, Latiff NAA, Nasirudeen AMA (2014). Factors contributing to academic performance of students in a Tertiary Institution in Singapore. Am J Educational Res.

[2] Ritchie SJ, Bates TC (2013). Enduring links from childhood mathematics and reading achievement to adult socioeconomic status. Psychol Sci.

[3] Mould T, DeLoach SB, Moving Beyond GPA. Alternative measures of success and predictive factors in Honors Programs. J Natl Collegiate Honors Council. 2017.

[4] Ong CKY, Hutchesson MJ, Patterson AJ, Whatnall MC (2021). Is there an association between Health Risk Behaviours and Academic Achievement among University students?. Int J Environ Res Public Health.

[5] Engin-Demir C (2009). Factors influencing the academic achievement of the turkish urban poor. Int J Educational Dev.

[6] Moghadari-Koosha M, Moghadasi-Amiri M, Cheraghi F, Mozafari H, Imani B, Zandieh M, Self-Efficacy (2020). Self-regulated learning, and motivation as factors influencing academic achievement among paramedical students: a correlation study. J Allied Health.

[7] Nabizadeh S, Hajian S, Sheikhan Z, Rafiei F (2019). Prediction of academic achievement based on learning strategies and outcome expectations among medical students. BMC Med Educ.

[8] Zheng B, Chang C, Lin C-H, Zhang Y, Self-Efficacy (2021). Academic motivation, and Self-Regulation: how do they predict academic achievement for medical students?. Med Sci Educ.

[9] Pinquart M, Kauser R (2018). Do the associations of parenting styles with behavior problems and academic achievement vary by culture? Results from a meta-analysis. Cultur Divers Ethnic Minor Psychol.

[10] Adeyemi AJ, Lasisi OI, Ojile P, Abdulkadir M (2020). The effect of furniture intervention on the occurrence of musculoskeletal disorders and academic performance of students in North-West Nigeria. Work.

[11] Dyer AM, Kristjansson AL, Mann MJ, Smith ML, Allegrante JP (2017). Sport participation and academic achievement: a longitudinal study. Am J Health Behav.

[12] Worsley JD, Harrison P, Corcoran R. Bridging the gap: exploring the Unique Transition from Home, School or College Into University. Front Public Health. 2021;9. 10.3389/fpubh.2021.634285 PMC8009977 33816421

[13] Riordan BC, Carey KB (2019). Wonderland and the rabbit hole: a commentary on university students’ alcohol use during first year and the early transition to university. Drug Alcohol Rev.

[14] Briggs ARJ, Clark J, Hall I (2012). Building bridges: understanding student transition to university. Qual High Educ.

[15] Porru F, Schuring M, Bültmann U, Portoghese I, Burdorf A, Robroek SJW (2022). Associations of university student life challenges with mental health and self-rated health: a longitudinal study with 6 months follow-up. J Affect Disord.

[16] Åsberg K, Eldh AC, Löf M, Bendtsen M (2022). A balancing act-finding one´s way to health and well-being: a qualitative analysis of interviews with swedish university students on lifestyle and behavior change. PLoS ONE.

[17] Alzahrani SH, Saeedi AA, Baamer MK, Shalabi AF, Alzahrani AM (2020). Eating Habits among Medical students at King Abdulaziz University, Jeddah, Saudi Arabia. Int J Gen Med.

[18] El-Ammari A, Kazdouh HE, Bouftini S, Fakir SE, Achhab YE (2020). Social-ecological influences on unhealthy dietary behaviours among moroccan adolescents: a mixed-methods study. Public Health Nutr.

[19] Alolabi H, Alchallah MO, Mohsen F, Marrawi M, Alourfi Z (2022). Social and psychosocial factors affecting eating habits among students studying at the syrian private University: a questionnaire based cross-sectional study. Heliyon.

[20] Naja F, Hwalla N, Itani L, Karam S, Mehio Sibai A, Nasreddine L (2015). A western dietary pattern is associated with overweight and obesity in a national sample of lebanese adolescents (13–19 years): a cross-sectional study. Br J Nutr.

[21] Yahia N, Achkar A, Abdallah A, Rizk S (2008). Eating habits and obesity among lebanese university students. Nutr J.

[22] Shatwan IM, Aljefree NM, Almoraie NM (2022). Snacking pattern of college students in Saudi Arabia: a cross-sectional study. BMC Nutr.

[23] Ghozayel M, Ghaddar A, Farhat G, Nasreddine L, Kara J, Jomaa L (2020). Energy drinks consumption and perceptions among University students in Beirut, Lebanon: a mixed methods approach. PLoS ONE.

[24] Suardiaz-Muro M, Morante-Ruiz M, Ortega-Moreno M, Ruiz MA, Martín-Plasencia P, Vela-Bueno A (2020). [Sleep and academic performance in university students: a systematic review]. Rev Neurol.

[25] Reuter PR, Forster BL, Brister SR (2021). The influence of eating habits on the academic performance of university students. J Am Coll Health.

[26] Marchand NE, Jensen MK (2017). The role of Dietary and Lifestyle factors in maintaining Cognitive Health. Am J Lifestyle Med.

[27] Mintzer J, Donovan KA, Kindy AZ, Lock SL, Chura LR, Barracca N (2019). Lifestyle choices and Brain Health. Front Med (Lausanne).

[28] Sogari G, Velez-Argumedo C, Gómez MI, Mora C (2018). College Students and Eating Habits: a study using an ecological model for healthy behavior. Nutrients.

[29] Whatnall MC, Patterson AJ, Burrows TL, Hutchesson MJ (2019). Higher diet quality in university students is associated with higher academic achievement: a cross-sectional study. J Hum Nutr Diet.

[30] Burrows T, Goldman S, Pursey K, Lim R (2017). Is there an association between dietary intake and academic achievement: a systematic review. J Hum Nutr Diet.

[31] Faught EL, Qian W, Carson VL, Storey KE, Faulkner G, Veugelers PJ (2019). The longitudinal impact of diet, physical activity, sleep, and screen time on canadian adolescents’ academic achievement: an analysis from the COMPASS study. Prev Med.

[32] Correa-Burrows P, Rodríguez Y, Blanco E, Gahagan S, Burrows R (2017). Snacking quality is Associated with secondary School Academic Achievement and the intention to Enroll in Higher Education: a cross-sectional study in adolescents from Santiago, Chile. Nutrients.

[33] Spencer SJ, Korosi A, Layé S, Shukitt-Hale B, Barrientos RM (2017). Food for thought: how nutrition impacts cognition and emotion. NPJ Sci Food.

[34] Naveed S, Lakka T, Haapala EA (2020). An overview on the Associations between Health Behaviors and Brain Health in Children and Adolescents with Special Reference to Diet Quality. Int J Environ Res Public Health.

[35] Sohayla-M.-Attalla, Sakinah-Ruhi, Che-Nur-Fadhlina-Bt-Che-Mud. Effect of cigarette smoking on the academic achievement among management and Science University students. Malaysian J Med Health Sci. 2020;:18–22.

[36] Sun J, Buys N, Stewart D, Shum D, Farquhar L (2011). Smoking in australian university students and its association with socio-demographic factors, stress, health status, coping strategies, and attitude. Health Educ.

[37] Pennanen M, Haukkala A, De Vries H, Vartiainen E (2011). Academic achievement and smoking: is self-efficacy an important factor in understanding social inequalities in finnish adolescents?. Scand J Public Health.

[38] Dearfield CT, Chen-Sankey JC, McNeel TS, Bernat DH, Choi K (2021). E-cigarette initiation predicts subsequent academic performance among youth: results from the PATH study. Prev Med.

[39] Kinnunen JM, Lindfors P, Rimpelä A, Salmela-Aro K, Rathmann K, Perelman J (2016). Academic well-being and smoking among 14- to 17-year-old schoolchildren in six european cities. J Adolesc.

[40] Stea TH, Knutsen T, Torstveit MK (2014). Association between short time in bed, health-risk behaviors and poor academic achievement among norwegian adolescents. Sleep Med.

[41] Seoane HA, Moschetto L, Orliacq F, Orliacq J, Serrano E, Cazenave MI (2020). Sleep disruption in medicine students and its relationship with impaired academic performance: a systematic review and meta-analysis. Sleep Med Rev.

[42] Ramar K, Malhotra RK, Carden KA, Martin JL, Abbasi -Feinberg, Fariha, Aurora RN et al. Sleep is essential to health: an American Academy of Sleep Medicine position statement. J Clin Sleep Med 17:2115–9. 10.5664/jcsm.9476 PMC8494094 34170250

[43] Donnelly JE, Hillman CH, Greene JL, Hansen DM, Gibson CA, Sullivan DK (2017). Physical activity and academic achievement across the curriculum: results from a 3-year cluster-randomized trial. Prev Med.

[44] Ardila CM, Gómez-Restrepo ÁM (2021). Relationship between physical activity, academic achievement, gender, and learning styles in students of a latin american Dental School: a cross-sectional study. J Educ Health Promot.

[45] Al-Drees A, Abdulghani H, Irshad M, Baqays AA, Al-Zhrani AA, Alshammari SA (2016). Physical activity and academic achievement among the medical students: a cross-sectional study. Med Teach.

[46] Depression. https://www.who.int/news-room/fact-sheets/detail/depression. Accessed 14 Jan 2023.

[47] Eisenberg D, Gollust SE, Golberstein E, Hefner JL (2007). Prevalence and correlates of depression, anxiety, and suicidality among university students. Am J Orthopsychiatry.

[48] Gao L, Xie Y, Jia C, Wang W (2020). Prevalence of depression among chinese university students: a systematic review and meta-analysis. Sci Rep.

[49] Ibrahim AK, Kelly SJ, Adams CE, Glazebrook C (2013). A systematic review of studies of depression prevalence in university students. J Psychiatr Res.

[50] Hammoudi Halat D, Younes S, Safwan J, Akiki Z, Akel M, Rahal M (2022). Pharmacy students’ Mental Health and Resilience in COVID-19: an Assessment after one year of Online Education. Eur J Investig Health Psychol Educ.

[51] Liu W, Sun N, Guo J, Zheng Z (2022). Campus Green Spaces, academic achievement and Mental Health of College Students. Int J Environ Res Public Health.

[52] Mahdavi P, Valibeygi A, Moradi M, Sadeghi S. Relationship between achievement motivation, Mental Health and Academic Success in University students. Int Q Community Health Educ. 2021;:272684X211025932. 10.1177/0272684X211025932 34176355

[53] Gedeon, R., Hallit, S. & Wakim, L.H. Food insecurity and eating habits of Lebanese children aged 5–11 years during the COVID-19 pandemic and the socioeconomic crisis: a national study. BMC Public Health 22, 1982 (2022). 10.1186/s12889-022-14387-z. 10.1186/s12889-022-14387-z PMC9616611 36307806

[54] Abdul-Reda Abourjeili S, Harb S. The deteriorated Educational reality in Lebanon: towards “Another” critical Approach. Arab Reform Initiative; 2020.

[55] Itani R, Mattar L, Kharroubi S, Bosqui T, Diab-El-Harake M, Jomaa L (2022). Food insecurity and mental health of college students in Lebanon: a cross-sectional study. J Nutr Sci.

[56] El Othman R, Touma E, El Othman R, Haddad C, Hallit R, Obeid S (2021). COVID-19 pandemic and mental health in Lebanon: a cross-sectional study. Int J Psychiatry Clin Pract.

[57] Hammoudi Halat HH, Dalal, Marwan A, Fatima H, Hassan H, Rawad K, Ehsan S-A (2020). Insights into the positive role of a Higher Education Institution in the Prevention of Misinformation during Pandemics: the Health Committee Model during COVID-19. Coronaviruses.

[58] Cuschieri S (2019). The STROBE guidelines. Saudi J Anaesth.

[59] Yun TC, Ahmad SR, Quee DKS (2018). Dietary Habits and Lifestyle Practices among University students in Universiti Brunei Darussalam. Malays J Med Sci.

[60] Gomez-Pinilla F, Gomez AG (2011). The influence of dietary factors in Central Nervous System Plasticity and Injury Recovery. PM R.

[61] Alzyoud S, Weglicki LS, Kheirallah KA, Haddad L, Alhawamdeh KA (2013). Waterpipe smoking among middle and high school jordanian students: patterns and predictors. Int J Environ Res Public Health.

[62] Weary-Smith KA. Validation of the physical activity index (PAI) as a measure of total activity load and total kilocalorie expenditure during submaximal treadmill walking. 2007. 10.1123/jpah.9.6.757 21952161

[63] Stadler M, Kemper CJ, Greiff S (2021). Assessing subjective university success with the subjective academic achievement Scale (SAAS). Eur Educational Researcher.

[64] Ali AM, Alameri RA, Hendawy AO, Al-Amer R, Shahrour G, Ali EM (2022). Psychometric evaluation of the depression anxiety stress scale 8-items (DASS-8)/DASS-12/DASS-21 among family caregivers of patients with dementia. Front Public Health.

[65] A Primer on Partial Least Squares Structural Equation Modeling (PLS-SEM). SAGE Publications Inc. 2023. https://us.sagepub.com/en-us/nam/a-primer-on-partial-least-squares-structural-equation-modeling-pls-sem/book244583. Accessed 13 Jan 2023.

[66] Tadese M, Yeshaneh A, Mulu GB (2022). Determinants of good academic performance among university students in Ethiopia: a cross-sectional study. BMC Med Educ.

[67] Mehare T, Kassa R, Mekuriaw B, Mengesha T (2020). Assessing predictors of academic performance for NMEI curriculum-based medical students found in the Southern Ethiopia. Educ Res Int.

[68] Burrows TL, Whatnall MC, Patterson AJ, Hutchesson MJ (2017). Associations between Dietary Intake and Academic Achievement in College students: a systematic review. Healthc (Basel).

[69] Gao CL, Zhao N, Shu P. Breakfast consumption and academic achievement among chinese adolescents: a Moderated Mediation Model. Front Psychol. 2021;12. 10.3389/fpsyg.2021.700989 PMC8647908 34880802

[70] Adolphus K, Lawton CL, Dye L (2013). The effects of breakfast on behavior and academic performance in children and adolescents. Front Hum Neurosci.

[71] Verulava T, Devnozashvili R (2021). Nutrition and academic performance among adolescences. Romanian J Diabetes Nutr Metabolic Dis.

[72] O’Neil CE, Byrd-Bredbenner C, Hayes D, Jana L, Klinger SE, Stephenson-Martin S (2014). The role of breakfast in Health: definition and criteria for a quality breakfast. J Acad Nutr Dietetics.

[73] Galioto R, Spitznagel MB (2016). The Effects of Breakfast and Breakfast Composition on Cognition in Adults123. Adv Nutr.

[74] Wengreen HJ, Moncur C (2009). Change in diet, physical activity, and body weight among young-adults during the transition from high school to college. Nutr J.

[75] Deliens T, Clarys P, De Bourdeaudhuij I, Deforche B (2013). Weight, socio-demographics, and health behaviour related correlates of academic performance in first year university students. Nutr J.

[76] Logi Kristjánsson Á, Dóra Sigfúsdóttir I, Allegrante JP (2010). Health Behavior and Academic Achievement among Adolescents: the relative contribution of Dietary Habits, physical activity, body Mass Index, and self-esteem. Health Educ Behav.

[77] Qasrawi R, Halawa DAA, Ayyad R, Sabah HA, Taweel H, Abdeen Z. Links between nutrition, life style habits and academic achievement in palestinian schoolchildren: a cross-sectional study. Al-Quds J Nat Sci. 2021;1.

[78] Purtell KM, Gershoff ET (2015). Fast food consumption and academic growth in late childhood. Clin Pediatr (Phila).

[79] Li X, Braakhuis A, Li Z, Roy R. How does the University Food Environment Impact Student Dietary Behaviors? A systematic review. Front Nutr. 2022;9. 10.3389/fnut.2022.840818 PMC9090611 35571951

[80] Kim SY, Sim S, Park B, Kong IG, Kim J-H, Choi HG (2016). Dietary Habits are Associated With School performance in adolescents. Med (Baltim).

[81] Correa-Burrows P, Burrows R, Blanco E, Reyes M, Gahagan S (2016). Nutritional quality of diet and academic performance in chilean students. Bull World Health Organ.

[82] Gómez-Pinilla F (2008). Brain foods: the effects of nutrients on brain function. Nat Rev Neurosci.

[83] El-Kassas G. Obesity Risk Factors among Beirut Arab University Students in Tripoli-Lebanon. 2015;5.

[84] Hafez SM, Ghazawy ER, Mahfouz EM, Abd-Elrahman A, Emam SA (2022). Obesity/Overweight among University students.

[85] Boukrim M, Obtel M, Lahlou L, Razine R (2021). University students’ perceptions and factors contributing to obesity and overweigh in Southern of Morocco. Afr Health Sci.

[86] Makkawy E, Alrakha AM, Al-Mubarak AF, Alotaibi HT, Alotaibi NT, Alasmari AA (2021). Prevalence of overweight and obesity and their associated factors among health sciences college students, Saudi Arabia. J Family Med Prim Care.

[87] Alhazmi A, Aziz F, Hawash MM (2021). Association of BMI, physical activity with academic performance among female students of Health Colleges of King Khalid University, Saudi Arabia. Int J Environ Res Public Health.

[88] Al-Mukhtar R, Musaiger A (2000). Obesity in female students in the United Arab Emirates University. Bahrain Med Bull.

[89] Fedewa MV, Das BM, Evans EM, Dishman RK (2014). Change in weight and adiposity in college students: a systematic review and meta-analysis. Am J Prev Med.

[90] Vella-Zarb RA, Elgar FJ (2009). The “freshman 5”: a meta-analysis of weight gain in the freshman year of college. J Am Coll Health.

[91] Al Qauhiz NM (2010). Obesity among saudi female University students: Dietary Habits and Health Behaviors. J Egypt Public Health Assoc.

[92] H A-K MW. R Y. Trends of obesity and overweight among College students in Oman: a cross sectional study. Sultan Qaboos University medical journal. 2012;12. 10.12816/0003090 PMC3286720 22375261

[93] Peltzer K, Pengpid S, Samuels TA, Özcan NK, Mantilla C, Rahamefy OH (2014). Prevalence of Overweight/Obesity and its Associated factors among University students from 22 countries. Int J Environ Res Public Health.

[94] Barrigas C, Fragoso I (2012). Obesity, academic performance and reasoning ability in portuguese students between 6 and 12 years old. J Biosoc Sci.

[95] Alswat KA, Al-Shehri AD, Aljuaid TA, Alzaidi BA, Alasmari HD (2017). The association between body mass index and academic performance. Saudi Med J.

[96] Almarshad F, Does BMI. Affect Academic Performance? Study among Undergraduate Medical Students of Saudi Arabia and India. 2020.

[97] Shi Y, Yu H, Di S, Ma C. Body Mass Index and academic achievement among chinese secondary school students: the mediating effect of Inhibitory Control and the moderating effect of Social Support. Front Psychol. 2022;13. 10.3389/fpsyg.2022.835171 PMC8899538 35265020

[98] He J, Chen X, Fan X, Cai Z, Huang F (2019). Is there a relationship between body mass index and academic achievement? A meta-analysis. Public Health.

[99] Finn KE, Faith MS, Seo YS (2018). School Engagement in Relation to Body Mass Index and School Achievement in a high-school age sample. J Obes.

[100] Tucktuck M, Ghandour R, Abu-Rmeileh NME (2017). Waterpipe and cigarette tobacco smoking among palestinian university students: a cross-sectional study. BMC Public Health.

[101] Nasser AMA, Geng Y, Al-Wesabi SA (2020). The prevalence of smoking (cigarette and waterpipe) among University students in some Arab Countries: a systematic review. Asian Pac J Cancer Prev.

[102] Nakkash R, Khader Y, Chalak A, Abla R, Abu-Rmeileh NME, Mostafa A (2022). Prevalence of cigarette and waterpipe tobacco smoking among adults in three Eastern Mediterranean countries: a cross-sectional household survey. BMJ Open.

[103] Hallit S, Obeid S, Sacre H, Salameh P (2021). Lebanese waterpipe dependence scale (LWDS-11) validation in a sample of lebanese adolescents. BMC Public Health.

[104] Akel M, Sakr F, Fahs I, Dimassi A, Dabbous M, Ehlinger V (2022). Smoking behavior among adolescents: the lebanese experience with cigarette smoking and Waterpipe Use. Int J Environ Res Public Health.

[105] Jawad M, McEwen A, McNeill A, Shahab L (2013). To what extent should waterpipe tobacco smoking become a public health priority?. Addiction.

[106] Primack BA, Shensa A, Kim KH, Carroll MV, Hoban MT, Leino EV (2013). Waterpipe smoking among U.S. university students. Nicotine Tob Res.

[107] Pennanen M, Haukkala A, de Vries H, Vartiainen E (2011). Longitudinal study of relations between school achievement and smoking behavior among secondary school students in Finland: results of the ESFA study. Subst Use Misuse.

[108] Busch V, Loyen A, Lodder M, Schrijvers AJP, van Yperen TA, de Leeuw JRJ (2014). The Effects of Adolescent Health-Related behavior on academic performance: a systematic review of the Longitudinal evidence. Rev Educ Res.

[109] Basch CE (2011). Healthier students are better Learners: a missing link in School Reforms to close the achievement gap. J Sch Health.

[110] Al-Drees A, Abdulghani H, Irshad M, Baqays AA, Al-Zhrani AA, Alshammari SA (2016). Physical activity and academic achievement among the medical students: a cross-sectional study. Med Teach.

[111] Lipošek S, Planinšec J, Leskošek B, Pajtler A, PHYSICAL ACTIVITY OF, UNIVERSITY STUDENTS AND ITS RELATION TO PHYSICAL FITNESS AND ACADEMIC SUCCESS (2018). Ann Kinesiologiae.

[112] Felez-Nobrega M, Hillman CH, Dowd KP, Cirera E, Puig-Ribera A (2018). ActivPAL^™^ determined sedentary behaviour, physical activity and academic achievement in college students. J Sports Sci.

[113] Lopes L, Santos R, Mota J, Pereira B, Lopes V (2017). Objectively measured sedentary time and academic achievement in schoolchildren. J Sports Sci.

[114] Chung Q-E, Abdulrahman SA, Khan MKJ, Sathik HBJ, Rashid A (2018). The relationship between levels of physical activity and academic achievement among medical and Health Sciences students at Cyberjaya University College of Medical Sciences. Malays J Med Sci.

[115] Cotman CW, Berchtold NC, Christie L-A (2007). Exercise builds brain health: key roles of growth factor cascades and inflammation. Trends Neurosci.

[116] Gomez-Pinilla F, Zhuang Y, Feng J, Ying Z, Fan G (2011). Exercise impacts brain-derived neurotrophic factor plasticity by engaging mechanisms of epigenetic regulation. Eur J Neurosci.

[117] Matthews VB, Aström M-B, Chan MHS, Bruce CR, Krabbe KS, Prelovsek O (2009). Brain-derived neurotrophic factor is produced by skeletal muscle cells in response to contraction and enhances fat oxidation via activation of AMP-activated protein kinase. Diabetologia.

[118] Vivar C, Potter MC, van Praag H (2013). All about running: synaptic plasticity, growth factors and adult hippocampal neurogenesis. Curr Top Behav Neurosci.

[119] Granieri A, Franzoi IG, Chung MC, Editorial. Psychological Distress Among University Students. Frontiers in Psychology. 2021;12. 10.3389/fpsyg.2021.647940 PMC8019774 33828513

[120] Jaisoorya T, Reddy YJ, Nair BS, Rani A, Menon PG, Revamma M (2017). Prevalence and correlates of obsessive-compulsive disorder and subthreshold obsessive-compulsive disorder among college students in Kerala, India. Indian J psychiatry.

[121] Ishii T, Tachikawa H, Shiratori Y, Hori T, Aiba M, Kuga K (2018). What kinds of factors affect the academic outcomes of university students with mental disorders? A retrospective study based on medical records. Asian J psychiatry.

[122] Nieuwoudt JE (2021). Psychological distress among students in Enabling Education: an exploratory study. Australian J Adult Learn.

[123] Yamada Y, Klugar M, Ivanova K, Oborna I (2014). Psychological distress and academic self-perception among international medical students: the role of peer social support. BMC Med Educ.

[124] Thompson M, Her P, Fetter AK, Perez J (2019). College Student Psychological Distress: relationship to Self-Esteem and Career decision self‐efficacy beliefs. Career Dev Q.

[125] Farran N (2021). Mental health in Lebanon: tomorrow’s silent epidemic. Ment Health Prev.

[126] Younes S, Safwan J, Rahal M, Hammoudi D, Akiki Z, Akel M (2022). Effect of COVID-19 on mental health among the young population in Lebanon. Encephale.

